# What place for virtual reality in the intensive care unit during medical procedures?

**DOI:** 10.1186/s40560-021-00545-9

**Published:** 2021-03-26

**Authors:** Floriane Puel, Vincent Minville, Fanny Vardon-Bounes

**Affiliations:** 1 Anesthesiology and Critical Care Unit, Toulouse University Hospital, 1 av du Pr Jean Poulhès, 31400 Toulouse, France; 2 RESTORE UMR 1301, Inserm 5070 CNRS, Paul Sabatier University, Toulouse, France; 3 INSERM U1297, Paul Sabatier University, Toulouse, France; 4 Toulouse University Hospital, 1 av du Pr J Poulhès, 31400 Toulouse, France

**Keywords:** Virtual reality, ICU, Pain, Anxiety

## Abstract

In the intensive care unit, patients are subject to discomforts and pain. Their management is essentially based on pharmacologic approaches. Immersive virtual reality could represent an adjunctive non-invasive and non-pharmacological pain control technique. It is based on real-time interaction with an artificial 360° immersive world using interfaces that enable physical and emotional perceptions to make the user feel better trying to reduce pain perception and to limit anxiety. Evaluation of virtual reality in intensive care unit is lacking and further studies are necessary before to introduce this alternative method for critical patients.

In the intensive care unit (ICU), patients are subject to discomforts such as aggressive noises, lights, and pain which can be perceived as a hostile environment. Anxiety and discomfort generated by repeated medical cares and invasive procedures can induce cognitive disorders in critical illness survivors [1]. Depression and post-traumatic stress disorder (PTSD) (incidence between 25 and 44%) [1, 2] are an integral part of the so-called post-intensive care syndrome (PICS) [3]. The PICS is increased by memories of frightening ICU experiences and is known to be associated with an increased morbidity-mortality and an impaired quality of life [2]. Physicians have recently started using immersive virtual reality (VR) as an adjunctive non-invasive and non-pharmacological pain control and anxiolysis technique. VR is based on real-time interaction with an artificial 360° immersive world; the patient can experience combinations of visual and auditory stimuli that help to immersing himself into the computer-generated reality and create a sense of presence within the environment.

According to the “gate control” theory [4], higher order thought processes, by closing the nerve gates to painful input, can change how the patient interprets incoming pain signals and can even change the amount of pain signals allowed to enter the brain. Thus, the hypothesis is that VR, by providing multisensory input, deflects attention into the virtual world and away from the pain. In 1999, Eccleston et al. theorized that any other attentional distraction may be placed in competition with the noxious stimulus and may inhibit interruption by pain [5]. This theory was strengthened by Hoffman et al. who used the combination of VR and brain fMRI (functional magnetic resonance imaging) to study the impact of VR on eight brain activity subjects during a painful thermal stimuli [6]. VR significantly reduced subjective reports of time spent thinking about pain by 44%, emotional pain by 45% and sensory pain ratings by 30% and can significantly reduce pain-related brain activity in five brain regions associated with emotional and sensory component of pain [6]. Since then, the literature on VR and pain management has increased. A randomized control trial showed that VR significantly reduced pediatric acute procedural pain and anxiety during a blood draw as compared with standard care [7]*.* Patients’ pain ratings during burn wound care significantly dropped when patients were distracted by immersive VR [8]. A meta-analysis suggested a positive effect on pain of VR *versus* standard care in 16 randomized controlled trials in acutely painful procedures although the strength of this finding was limited by clinical and statistical heterogeneity [9].

Although research on VR as a treatment intervention is on the rise, the literature on this topic in the adult ICU is quite sparse. In 2019, Blair et al. reported the case of a ventilated female on veno-venous extracorporeal membrane oxygenation (ECMO) placed under VR who subjectively reported decreased anxiety after each session [10] Gerber et al. demonstrated the feasibility of the use of VR for relaxation in the ICU and reported a significantly reduction of vital markers of physical stress (heart rate and blood pressure) in 37 patients [11]. Recently, literature showed that a VR meditative intervention improved patients’ experience in the ICU by reducing anxiety and depression [12] and by improving the quality of patients’ sleep [13]. The results appear promising since it showed that critically ill patients mostly considered the sessions enjoyable and relaxing [14].

To the best of our knowledge, no studies have examined VR technology to improve ICU patients experience during painful procedures. The management of acute pain related to healthcare interventions remains a major global challenge. In our view, it will be interesting to assess the use of VR during acute painful procedures or chronic daily use in the ICU adult patients, without neurologic disorder, ventilated or not. We assume that reducing excessive pain may also reduce the stress experienced by the patient, the patient’s family and the health care givers. It will be interesting to evaluate if VR, by creating a distraction from unavoidable discomforts, can reduce anxiety and pain. Further clinical studies with standardized protocols and a well-trained professional team are required to assess feasibility and safety of this technique. The objective is to propose an immersive 360° visual experience that offers an opportunity for patients to be active in choosing experiences to relax their mind and their body: the patient decides which natural scenes (such as mountains or lakes) and background sounds he wants and how long each session would last to immerse himself in the experience. In terms of safety, it is important to study any side effects that may occur during a session: tolerance of the headset isolation, neurocognitive (anxiety, agitation), or physical (nausea and vomiting, displacement of the medical device) effects and risk of cross infection between patients. The challenge for the future is to make VR accessible 24/7 to improve patients’ ICU experience and to introduce alternative methods for patients’ recovery.

## Data Availability

Not applicable

## Abbreviations

ICU: Intensive care unit
PTSD: Post-traumatic stress disorder
PICS: Post-intensive care syndrome
VR: Virtual reality
fMRI: Functional magnetic resonance imaging
ECMO: Extra corporeal membrane oxygenation
